# Identification of 3^′^ gene ends using transcriptional and genomic conservation across vertebrates

**DOI:** 10.1186/1471-2164-13-708

**Published:** 2012-12-18

**Authors:** Marcos Morgan, Alessandra Iaconcig, Andrés Fernando Muro

**Affiliations:** 1 International Centre for Genetic Engineering and Biotechnology (ICGEB), Padriciano 99, I 34149, Trieste, Italy; 2 Present address: EMBL Monterotondo Adriano Buzzati-Traverso Campus, Via Ramarini 32, 00015, Monterotondo, Italy

**Keywords:** pre-mRNA cleavage site, PhyloP, RNA sequencing, TransMap

## Abstract

**Background:**

In higher eukaryotes, gene expression is regulated at different levels. In particular, 3^′^UTRs play a central role in translation, stability and subcellular localization of transcripts. In recent years, the development of high throughput sequencing techniques has facilitated the acquisition of transcriptional data at a genome wide level. However, annotation of the 3^′^ ends of genes is still incomplete, thus limiting the interpretation of the data generated. For example, we have previously reported two different genes, *ADD2* and *CPEB3*, with conserved 3^′^UTR alternative isoforms not annotated in the current versions of Ensembl and RefSeq human databases.

**Results:**

In order to evaluate the existence of other conserved 3^′^ ends not annotated in these databases we have now used comparative genomics and transcriptomics across several vertebrate species. In general, we have observed that 3^′^UTR conservation is lost after the end of the mature transcript. Using this change in conservation before and after the 3^′^ end of the mature transcripts we have shown that many conserved ends were still not annotated. In addition, we used orthologous transcripts to predict 3^′^UTR extensions and validated these predictions using total RNA sequencing data. Finally, we used this method to identify not annotated 3^′^ ends in rats and dogs. As a result, we report several hundred novel 3^′^UTR extensions in rats and a few thousand in dogs.

**Conclusions:**

The methods presented here can efficiently facilitate the identification of not-yet-annotated conserved 3^′^UTR extensions. The application of these methods will increase the confidence of orthologous gene models across vertebrates.

## Background

Gene expression in higher eukaryotes is a complex phenomenon which is regulated at different levels. In particular, regulatory sequences present in the messenger RNAs (mRNAs) can determine their localization, stability and translational activity. Recent developments in high throughput sequencing have greatly facilitated the acquisition of transcriptional data [1-3]. Nowadays, it is possible to obtain the transcriptome landscape of a tissue or cell line in a few days at a relatively low cost. However, recent results obtained using total RNA sequencing from humans have shown that there are still thousands of not annotated exons or exon extensions [4,5] limiting the interpretation of the data generated.

Genome annotation is a rather complex process that can be facilitated with computer-based methods but ultimately requires extensive manual curation. In order to address this challenging problem, different consortia are trying to generate reference databases of different species transcriptomes. Two of the most popular and rather independent databases currently available for the human genome are the RefSeq and the Ensembl gene models [6,7]. These databases consider all types of long transcripts, including non-coding RNAs. They are of high quality as they have been extensively curated in the past and are periodically updated. In addition, they are freely available to the scientific community through accessible web interfaces.

One of the big hurdles in higher eukaryotes genome annotation is that genes generally encode for more than one transcript isoform. For example, the same gene can be transcribed from different promoters, which can be tissue-specific or activated during different phases of development. In addition, exons can be alternatively spliced to generate different mature isoforms. A third way in which different mature transcripts can originate from the same locus is by alternative usage of cleavage and polyadenylation sites, referred hereafter as polyadenylation sites (PAS). The PAS demarcates the end of the mature transcript and its usage is strongly determined by the presence of a polyA signal (hexanucleotide motif, HexM) and a Downstream Sequence Element (DSE) [8]. The canonical HexM is AAUAAA, but other variants such as AUUAAA are frequently used [9,10]. This hexanucleotide motif is located 15–25 nucleotides upstream of the end of the mature transcript whilst the DSE is a G/GU rich region located approximately 25 nucleotides downstream of the end of the mature transcripts. These signals are recognized by a well-described protein complex that cleaves the transcript precursor [11]. The cleavage is followed by the addition of a polyA tail at the free 3^′^ end.

Although the mechanisms that determine the promoter usage sites and alternative splice sites have been extensively studied much less is known about the alternative selection of PASs. Nevertheless, in recent years, alternative PAS usage was shown to be very common and tightly regulated during development [12,13]. Importantly, the selection of a particular PAS determines the length of the 3^′^ UnTranslated Regions (3^′^UTRs) of genes encoding for proteins and, consequently, the regulatory elements present in these sequences. In practice, the use of a PAS proximal to the stop codon generally gives rise to a short 3^′^UTR transcript whilst use of a more distal PAS generates a longer 3^′^UTR. The functional consequences of PAS-site choice is that distinct regulatory elements will be exclusively present in the longer isoform of the transcript as opposed to the shorter isoform. During the last decade it has become evident that the 3^′^UTRs of genes play a central role in gene regulation as they are the natural targets of miRNAs [14].

We previously reported two instances in which alternative 3^′^ ends of genes were not annotated in the available human genome databases. The first case corresponds to the *ADD2* gene [15]. This gene is mainly expressed in erythroid tissues and in the brain. In erythroid tissues, a specific promoter is used together with proximal PASs. On the other hand, in brain a different tissue specific promoter is used and a more distal PAS is recognized by the cleavage and polyadenylation machinery. As a result, the brain-specific *ADD2* transcript is 5–6 Kb longer than the erythropoietic isoform. This brain-specific site is still not annotated in databases although it corresponds to the most abundant *ADD2* mRNA species in brain, is highly expressed in mice and humans, and is conserved across vertebrates. The second case corresponds to the *CPEB3* transcript [16]. This gene is expressed in different tissues where usually a proximal PAS is used [17]. In brain, however, a more distal PAS is also used, that is abundantly expressed and extremely conserved. Again, this distal, brain specific transcript is still not annotated in the current databases.

Therefore, we believe that a more complete annotation of PASs is needed. To this end, we decided to look for evidence of other potential PASs that might not be annotated in the current versions of RefSeq and Ensembl gene predictions. In particular, we concentrated on putative evolutionarily conserved sites, similar to the ones that we previously described for the ADD2 and CPEB3 transcripts, as a strong selective pressure may reflect relevant biological functions. Using these criteria and combining them with transcriptional information from human Expressed Sequence Tags (ESTs), we identified several highly conserved, still not annotated PASs in these databases. We also used a complementary approach in which we evaluated putative PAS used in other species, but not necessarily highly conserved at the genomic level. In this case, we used deep sequencing data from total RNA of eight different human tissues as transcriptional evidence. Finally, we applied our method to identify novel 3^′^ ends in rats and dogs. As we identified hundreds of conserved not annotated PASs in these species we propose that our method can be used to improve the annotation of any mammalian genome.

## Results

### Genomic conservation decreases after the polyadenylation site (PAS)

It is well known that 3^′^UTRs can be highly conserved [18]. However, we have previously observed that this conservation resulted to be lost immediately downstream of the PAS for the CPEB transcripts [16] with just one exception. In *CPEB3*, in fact, we observed an island of conservation approximately 2 Kb downstream of the annotated PAS. Interestingly, the end of this island coincided with the end of a cluster of ESTs suggesting the existence of a novel PASs, that we later validated through different biochemical approaches. Similarly to CPEBs, we also observed an important drop in conservation after the distal PAS of the *ADD2* gene (data not shown), which was associated with a cluster of ESTs [15]. In this work, therefore, we thought that we could use a similar rationale to detect other not annotated distal PASs at a genome-wide level.

**Figure 1:**
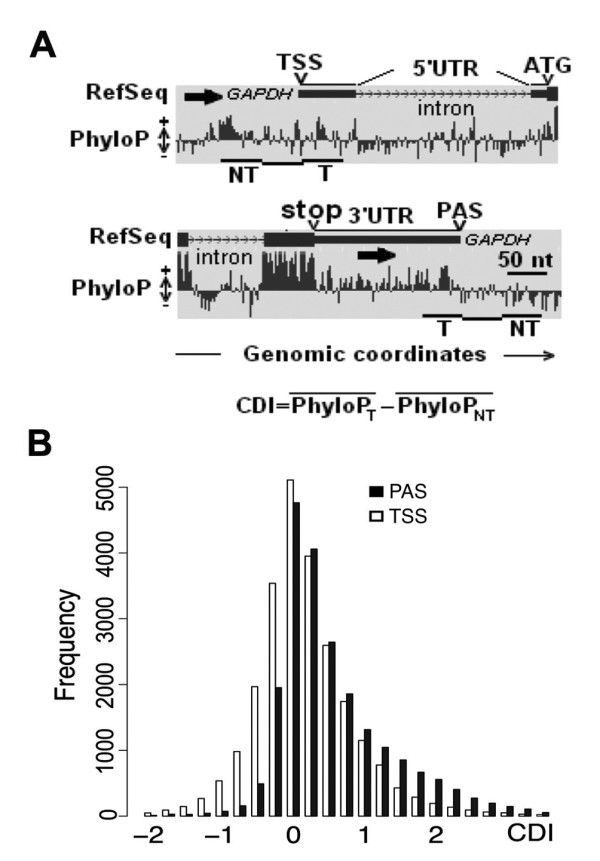
**Genomic conservation falls after the PAS.** Panel **A**. PhyloP conservation values for the genomic region spanning the first exon and first intron of a RefSeq annotated gene (*GAPDH*) (upper panel) and the last intron and the last exon of the same gene (lower panel). The windows were adapted from the UCSC genome browser [21]. TSS, Transcriptional Start Site; ATG, start codon; stop, stop codon; PAS, polyadenylation site; T, transcribed interval encoding for the mature transcript; NT, interval not encoding for the mature transcript; CDI, Conservation Drop Index. The black arrows indicate the direction of transcription of the gene. Panel **B**. Absolute frequency of TSS and PAS annotated in the RefSeq database according to their CDI.

To estimate the genomic conservation in regions associated with the transcripts’ extremes, we used PhyloP scores calculated for 44 vertebrates [19]. The PhyloP scores consider the conservation at the nucleotide level without taking into account the genomic context. To determine changes in conservation at the ends of each transcript we calculated the average PhyloP value of the 50 nucleotides present at the 3^′^ (or 5^′^) end of the mature transcript (“T”, see Figure 1A). We then calculated the average PhyloP value of a 50 nucleotide-long interval starting 50 nucleotides from the extreme of the mature transcript (“NT”, see Figure 1A). Finally, we defined a conservation drop index (CDI) as the difference between the average PhyloP values of the “T” region minus the average of the “NT” region. We considered 50 nucleotides upstream of the PAS in order to define an interval containing the well-conserved polyA signal towards the middle and then we also included a 50-nucleotide gap to account for the DSE. Therefore, a positive CDI value for the 3^′^ end indicates that the genomic region encoding the end of the mature transcript is more conserved than the adjacent downstream sequence. Instead, a CDI value close to 0 indicates that there are small differences in conservation between the mature transcript end and the adjacent downstream genomic sequence.

To determine whether the drop in conservation after the PAS was a general phenomenon, we first tested this hypothesis with the annotated genes present in the RefSeq database. To this end, we calculated the CDI for all the PASs annotated in RefSeq. As shown in Figure 1B, PASs had a positive CDI on average and the population was asymmetrically distributed towards positive values. This result indicated that many annotated PASs had an important drop in conservation like that observed for the CPEB genes [16]. In contrast, the distribution of transcriptional start sites (TSSs) did not have an extended tail towards the positive values (Figure 1B). This showed that the sharp drop in conservation observed in the 3^′^UTR was rarer at these ends of the transcript. Therefore, we postulated that this difference in conservation before and after the end of the mature transcript could be used to identify not annotated PASs but not TSSs.

### Genomic conservation sharply decreases in hundreds of putative PASs

**Figure 2:**
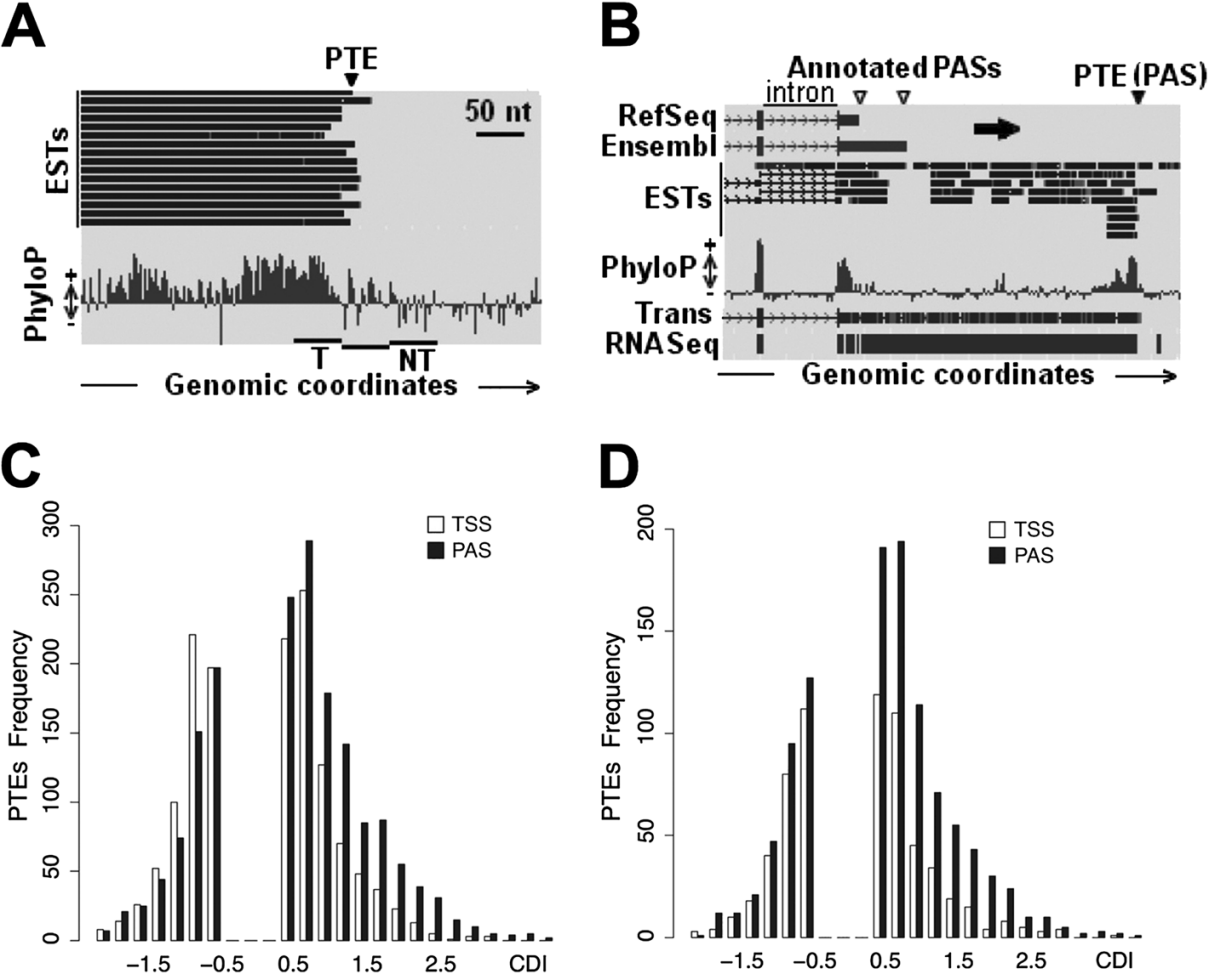
**Hundreds of putative PASs show a high CDI.** Panel **A**. PhyloP scores for the genomic region spanning the end of an EST cluster that originated a PTE (black arrowhead). The intervals used to calculate the PTE CDI are indicated below. T, transcribed interval encoding for the mature transcript; NT, interval not encoding for the mature transcript. Panel **B**. A PTE in a wider genomic context. The position of the PTE is indicated with a black arrowhead. The last two exons (and the last intron) of the most proximal RefSeq and Ensembl transcripts are shown above. Their direction of transcription is indicated by the black arrow. The positions of the annotated PASs are indicated with open arrowheads. The PTE was classified as a putative PAS for both RefSeq and Ensembl databases because the proximal reference transcripts are transcribed towards the PTE. Below a TransMap transcript that ends in the same position as the PTE is shown (Trans). In this case, the Trans transcript is supporting the PTE. On the bottom, the genomic region covered by total RNA deep sequencing reads (RNA seq). Panel **C**. Absolute frequency of PTEs, either putative PASs or putative TSS, according to their CDI. The PTEs were obtained using the RefSeq database as reference. Panel **D**. As in Panel C but using the Ensembl database as reference. PTEs with CDI in between −0.5 and +0.5 were omitted from the graphs shown in Panels C and D.

Next, to determine the presence of distal conserved PASs still not annotated in the RefSeq or Ensembl databases, we clustered ESTs finishing at the same position and assigned a CDI to each of them (Figure 2A). We refer to these clusters as Potential mature Transcript Extremes (PTEs). In addition, we classified each of these PTEs in putative TSSs or putative PASs according to the orientation of the most proximal reference gene (see Figure 2B, Materials and Methods Section, and Additional file 1: Figure S1). We did this analysis separately for the RefSeq and Ensembl gene predictions. We observed that, like for the annotated RefSeq genes, the putative PASs showed an asymmetric distribution towards high CDIs while this was less evident for TSSs (Figure 2C and D). In this way, the distribution of the putative PASs and TSSs resembled the distribution of the annotated ones, suggesting that many PTEs may correspond to real transcript extremes.

### Determination of the methods’ False Discovery Rate (FDR)

In order to evaluate the method, we then estimated the FDR at different CDI cutoffs. To determine whether a PTE was a true positive, we looked for transcripts on other databases with the predicted end. For example, we looked if the predicted ends not annotated in the RefSeq database were already annotated in the Ensembl database and vice versa.

Since predicted ends with high CDIs are highly conserved, we reasoned that orthologous ends are used in other species. Therefore, we also considered other species transcripts to estimate the FDR. In particular, we examined orthologous transcripts mapped with the TransMap program to the human genome [20].

To assess the FDR for a given CDI cutoff, we considered all PTEs with an equal or higher CDI. Next, for each of these predicted PASs, we assessed if they were represented or not on the above mentioned databases. The FDR was estimated as the number of PTEs not represented on other databases divided by the total number of PTEs.

**Figure 3:**
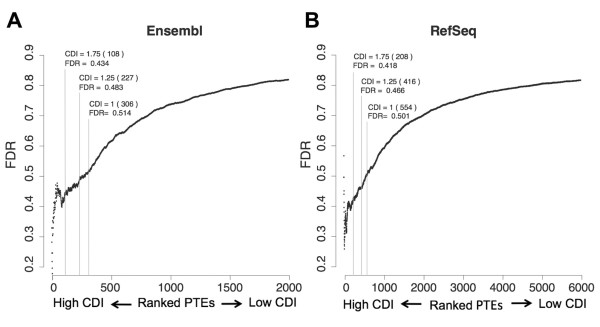
**PTEs with high CDI are more likely to be real PASs.** Panel **A**. FDR values calculated using each of the PTEs CDI as a cutoff. The PTEs were obtained using the Ensembl database as reference and only putative PASs were considered. PTEs were ranked according to their CDI. The PTE with the highest CDI is on the first position of the scale, the PTE with the second highest CDI is on the second position, and so on until the last position with the PTE with lowest CDI. Note that the spacing between contiguous PTEs is constant independently of the absolute difference of their CDI. The FDRs obtained using as cutoffs CDIs of 1, 1.25 and 1.75 are indicated. The numbers of PTEs with a CDI equal or higher than the cutoff used are shown between brackets. Panel **B**. As in A using the RefSeq database as reference to obtain the PTEs.

Given that every PTE has an associated CDI, we calculated the FDR using each of these CDIs as cutoffs. In this way, for every PTE we obtained an associated FDR as shown in Figure 3. For both Ensembl and RefSeq databases, we observed that the higher the CDI the lower the FDR. The FDR values for different cutoffs are similar for both databases but the total number of not annotated PASs is higher for the RefSeq database. For example, the FDRs for a CDI of 1.25 are of 0.483 and 0.466 for the Ensembl and RefSeq databases respectively while the numbers of PTEs with a CDI higher than 1.25 are 227 and 416. These results show that conservation signatures can be use to facilitate the identification of not annotated PASs.

Given that Ensembl annotation is more exhaustive than RefSeq, as it had a lower number of not annotated PASs, we used this database as reference for an exhaustive manual annotation. In order to confirm the predicted sites used in other species we performed a case-by-case examination of the putative PASs with CDI higher than 0.75, that were supported by an orthologous transcript (TransMap), an orthologous RefSeq transcript (Trans RefSeq), or a RefSeq transcript. To this end, we inspected the genomic context of the putative PASs using the graphical interface of the UCSC genome browser [21] and we confirmed that most of these sites appear to be functional, including the *ADD2* brain specific isoform (Additional file 2: Table S1) previously described [15].

We also found ~49 PTEs with CDI higher than 1 but without support from TransMap, Trans RefSeq or RefSeq transcripts, that could be functional PASs as well, including the previously characterized CPEB3 distal PAS [16]. On Additional file 3: Figure S2 we show examples of two of these not supported PTEs downstream of the annotated PASs of the *RNF130* and *RNF150* genes that could correspond to not annotated PASs. Taken together, these observations indicate that the above-calculated FDRs are overestimated.

The PhyloP conservation scores, used to calculate the CDI values, were calculated using 44 vertebrates’ genomes. These conservation scores, however, might be too stringent to detect PASs specific to a restricted group of vertebrates such as the mammals. In order to identify not annotated mammalian specific PASs, we repeated the analysis using PhyloP scores calculated across different mammals. We found a strong correlation between the vertebrate and mammalian specific CDIs (Additional file 4: Figure S3). However, we failed to detect any clear population of mammalian-specific PASs. Therefore, we conclude that the method detects mainly ancestral vertebrates 3^′^ ends.

### Identification of not annotated PASs using orthologous transcripts

**Figure 4:**
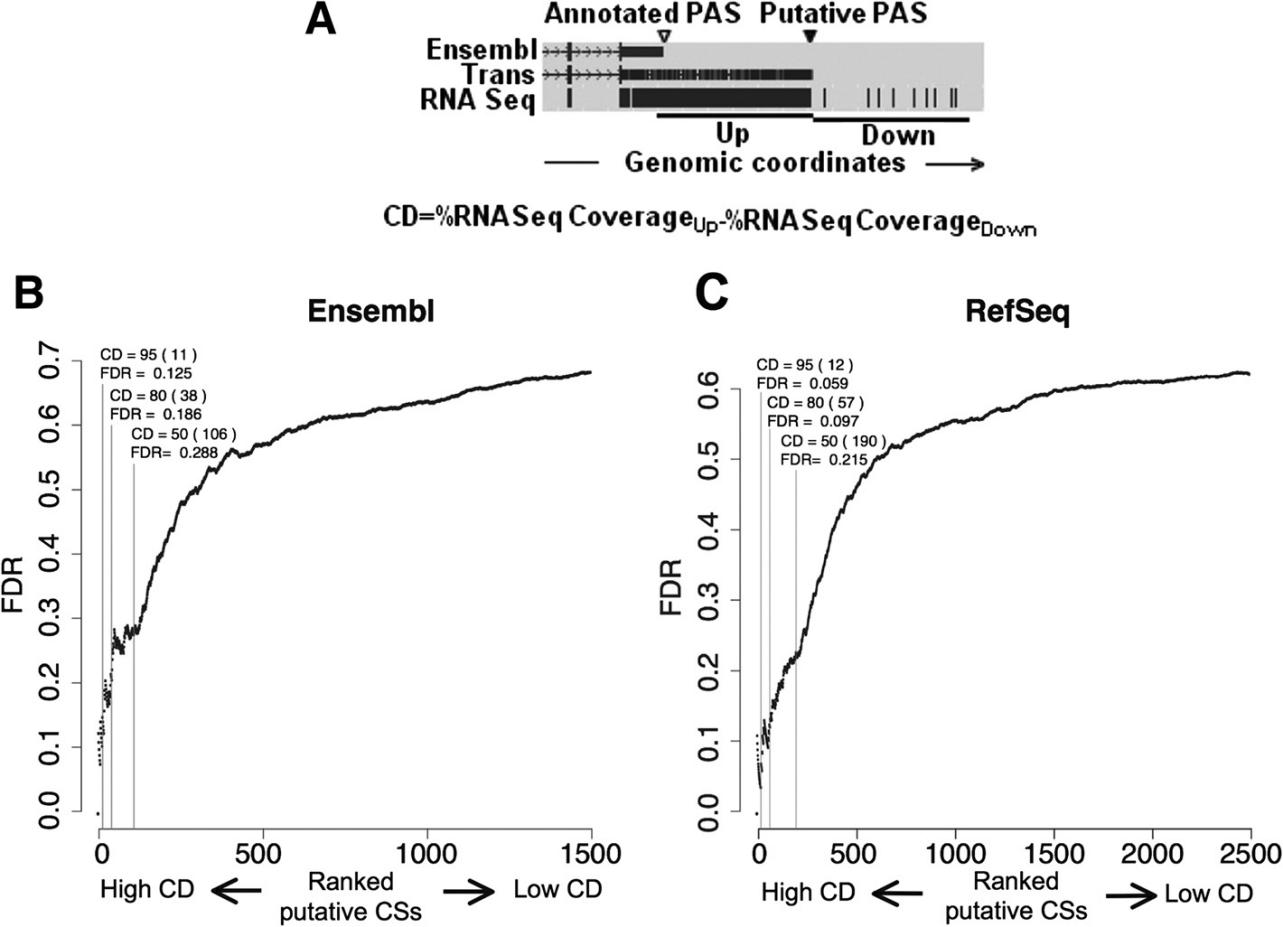
**Identification of PASs using orthologous transcripts and total RNA sequencing data.** Panel **A**. Genomic context of a putative PAS associated with a TransMap transcript (Trans). The last two exons (and the last intron) of the Ensembl prediction that could have a larger associated isoform using the putative PAS are shown above (Ensembl). The TransMap transcript and total RNA Seq coverage for the interval is shown below. The intervals used to calculate the Coverage Difference (CD) and the applied formula are indicated. Up, upstream interval; Down, downstream interval. Panel **B**. FDR values calculated using each of the putative PASs CD as a cutoff. The putative PASs were obtained using the Ensembl database as reference and were ranked according to their CD. The one with the highest CD is on the first position of the scale, the putative PASs with the second highest CD is on the second position, and so on until the last position with the putative PASs with lowest CD. The FDRs obtained using as cutoffs CDs of 95, 80 and 50 are indicated. The numbers of putative PASs with a CD equal or higher than the cutoff used are shown between brackets. Panel **C**. As in B using the RefSeq database as reference to obtain the putative PASs.

Next, we thought that several other conserved transcripts with not necessarily high CDI (CDI ≤0.75), might also not be correctly annotated. For this reason, in order to validate the usage of the PASs identified so far and to look for other not annotated ones, we designed a different approach using as transcriptional evidence deep sequencing reads, 32 nucleotide long, from human RNA produced by Wang and collaborators [2]. The strategy was based on the identification of orthologous transcripts with gene structures similar to those of the annotated genes but with longer 3^′^UTRs (see Figure 4A). Using these extensions as predictions, we calculated the read coverage percentage of the genomic interval spanning from the annotated PAS to the putative PAS (shown as “Up” in Figure 4A). We also calculated the read coverage percentage of an interval of the same length immediately downstream of the putative PAS (shown as “Down” in Figure 4A). We then defined the coverage difference (CD) as the difference between the reads coverage of the 3^′^UTR extension (Up) and the reads coverage of the interval immediately downstream (Down). Therefore, a CD value of 100 indicates that the proposed 3^′^UTR extension is completely covered by reads and no reads are found in an interval of the same length immediately downstream as expected in the presence of a strong PAS. Instead, a CD value of 0 indicates that there are no differences in the transcriptional levels upstream and downstream the putative PAS.

Using this approach, we detected several other not annotated PASs (See Additional file 5: Table S2 for a manually curated list of the sites identified, with CD higher than 50%, not annotated in the Ensembl database). We also calculated the FDR of the method as described before. The FDR increases as the CD decreases (Figure 4B and C) indicating that the method can facilitate the identification of not annotated PASs. We also confirmed with this approach that the Ensembl predictions are more complete than the RefSeq models. For example, the number of potential new PASs, with a CD higher than 50, was of 106 and 190 for Ensembl and RefSeq databases with FDR values of 0.288 and 0.215, respectively (Figure 4B and C).

### Extension of the method to other species

The development of deep sequencing techniques made possible to obtain a global and unbiased picture of the whole transcriptome with minimum effort and at a relatively low cost. In this way, new RNA-seq datasets from different organisms are being deposited every month in public repositories. Therefore, we envisioned that we could apply the previous method using the new information available to improve the annotation of other species. To this end, we analyzed different RNA-seq libraries from dogs and rats (see Materials and Methods Section).

**Figure 5:**
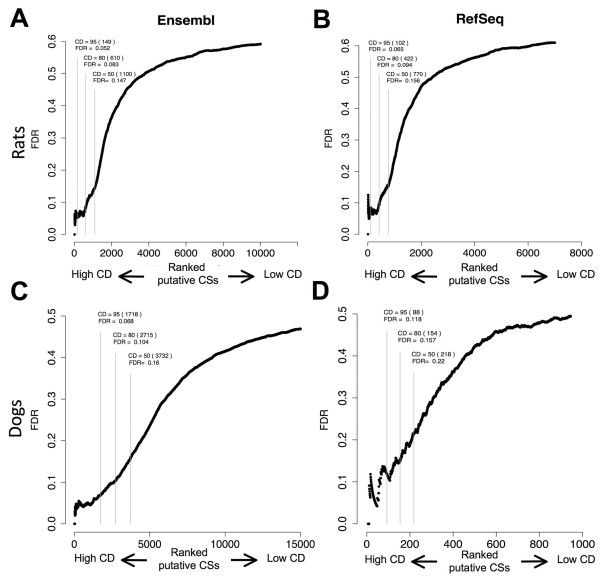
**Identification of PASs in rats and dogs using orthologous transcripts and total RNA sequencing data.** FDR values calculated using each of the putative PASs CD as a cutoff are shown as in Figure 4. The putative PASs were obtained using the Ensembl (Panels **A** and **C**) and RefSeq (Panels **B** and **D**) databases from rats (Panels **A** and **B**) and dogs (Panels **C** and **D**).

We first investigated the Ensembl and RefSeq models for rats. We found several hundred genes with potential gene extensions for both databases (Additional file 6: Tables S3A and S3B and see Additional file 7: Figure S4 for examples). To evaluate the performance of the method in this context, we calculated the FDR as we previously did while analyzing the human databases. As shown in Figures 5A and B, the FDR decreased for higher CD cutoffs, thus validating the use of this method on other species.

Next we investigated the RefSeq and Ensembl annotations of the dog genome. Using our method, we found a few thousand new potential PASs for the Ensembl models (Additional file 8: Table S4A and see Additional file 9: Figure S5 for examples). Instead, we identified very few new putative 3^′^ends for the RefSeq database in comparison (Additional file 8: Table S4B). The differences, in this case, can be explained by the limited number of gene models present on the RefSeq databases and not by a more exhaustive annotation of the RefSeq models (data not shown). Nevertheless, the FDR values again decreased for higher CD cutoffs (Figure 5C and D), showing that the method can be used even at early stages of the annotation process. In recent months we observed a major effort to improve the Ensembl models for dogs. To test whether our predictions were in agreement with the new models, we compared our proposed list of proposed gene ends with the PASs of the new transcripts. We observed that more than 500 of our predictions were incorporated into the new models (Additional file 8: Table S4A). This observation indicates that a significant proportion of our predictions are in agreement with the recently proposed models.

### Validation of PTEs by Northern blot analysis

**Figure 6:**
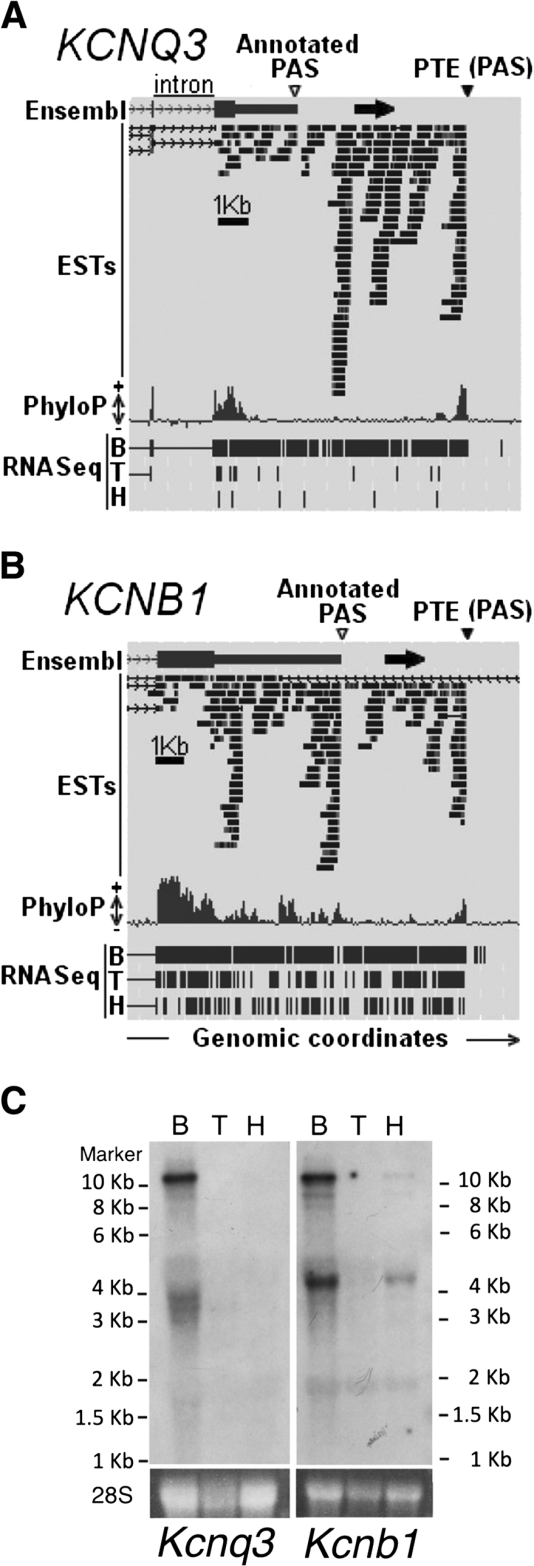
**Analyses of two mouse potassium channels transcripts, *Kcnb1 *and *Kcnq3, *with putative distal PASs.** Panel **A**. Genomic context of a PTE downstream of the *KCNQ3* gene. The interval shown goes from base 133,131,500 to base 133,145,500 of chromosome 8 of the human reference sequence (GRCh37/hg19). The PTE position is indicated with a black arrowhead. The black arrow indicates *KCNQ3* direction of transcription. The position of the annotated PAS is indicated with an open arrowhead. On the bottom, the genomic region covered by total RNA deep sequencing reads (RNA seq) from brain (B), testes (T) or heart (H). Panel **B**. As in Panel A, but for a PTE downstream of the *KCNB1* gene. The interval shown goes from base 47,978,000 to base 47,993,000 of chromosome 20. Panel **C**. Northern blot analysis of total RNA. Three different tissues were tested. B, brain; T, testes; H, heart. On the bottom of each panel, the 28S ribosomal RNA is shown as a loading control. The molecular weight is indicated. For *Kcnb1* mRNA, the expected size of the transcript that uses the annotated PAS is of ~3.7 Kb and the expected size of the transcript that uses the predicted distal PAS is of ~11.2 Kb. For *Kcnq3* mRNA, the expected size of the transcript that uses the annotated PAS is of ~2.9 Kb and the expected size of the transcript that uses the predicted distal PAS is of ~11.3 Kb.

In order to provide biochemical support for the bioinformatics results obtained so far, we decided to investigate some of the transcripts predicted to have long 3^′^UTRs by Northern blot analysis. As shown above, the use of a highly conserved PAS by any vertebrate is a good indication of the use of the orthologous site in humans. Thus, we selected the PTEs associated with the *KCNB1* and *KCNQ3* genes that were not annotated as PASs, neither in humans nor in mice Ensembl databases, and evaluated their expression in mice. In order to detect all the possible 3^′^UTR isoforms, we used probes proximal to the stop codon. In this way, we could simultaneously assess the use of the annotated isoforms, the predicted longer isoform, and any other intermediate species generated by alternative cleavage and polyadenylation. In parallel, we chose these genes because we noticed that they have an extensive coverage of deep sequencing reads obtained from brain RNA (Figures 6A and B). In addition, to investigate the tissue specificity of the genes, we used total RNA samples from brain, testes and heart.

As shown in Figure 6C, we observed for both genes a high molecular weight band of more than 10 Kbs consistent with the use of the not annotated PASs. As expected, we also observed high expression in brain but not in other tissues. Interestingly, the high molecular weight band was one of the main species observed in both cases. Similar results were previously reported by other groups for *KCNQ3* in mice and humans [22,23], further supporting our results.

In the latest versions of the Ensembl human annotations, the models have been merged with the manually annotated Havana transcripts. We observed that some of our predictions were incorporated into the unified database (Additional file 2: Table S1 and Additional file 5: Table S2). In most cases, the new gene ends did not correspond to 3^′^UTR extensions of the upstream transcripts but to novel large intervening non-coding RNAs (lincRNAs). This is the case for example of the PTE downstream the KCNB1 gene. Our experimental data (Figure 6) favor a model in which the PTE corresponds to the end of a 3^′^UTR extension of the KCNB1 gene, however, both models are not mutually exclusive.

In order to gain further evidence for the evolutionary conservation of the KCNB1 and KCNQ3 extended 3^′^UTRs, we investigated whether these isoforms were present in the corresponding rats and dogs ortholog genes. Although the annotated homologs in these species showed shorter 3^′^UTRs, the transcriptional evidence coming from RNA-Seq data supports the existence of the long isoforms (Additional file 10: Figure S6 and Additional file 11: Figure S7).

In conclusion, these observations provide evidence for the existence of long RNA species spanning the 3^′^UTR region linking the annotated transcripts with the PTEs. In addition, these results show that these not annotated species are abundantly expressed in the inspected tissues compared with the annotated ones. In agreement with this observation, the *kcnb1* predicted PAS corresponds to the 3^′^ end of the single *kcnb1* RefSeq model in mice. Similarly, the two RefSeq models of the gene in humans support the predicted *kcnq3* PAS.

## Discussion

After the completion of the human genome project, the annotation of the whole sequence became a major challenge. Most of the initial efforts were focused on the annotation of the coding sequences. Nowadays, however, increasing attention is also given to non-coding RNAs and untranslated regions. To achieve this aim, different methods have been developed in recent years to annotate some of these elements using deep sequencing data [24-26]. For example, a novel approach using histone methylation marks have been successfully used to identify large intervening non-coding RNAs (lincRNAs) [25]. In addition, important progresses have been made on 3^′^UTR annotation of model organisms, such as *Caenorhabditis elegans*, using high-throughput approaches [27,28]. However, we believe that 3^′^UTRs annotation in vertebrates also requires to be complemented with new and specific methods as the ones we described in this work.

In order to develop new appropriate approaches, different aspects of 3^′^ UTRs nature have to be considered. One particular hurdle of 3^′^UTR annotation is that the highly conserved elements that might be present in these sequences that could be used to define them can be separated from one another by other elements such as Alu sequences that are not necessarily conserved. Another difficulty is that untranslated regions, in particular 3^′^UTRs, can be several kilobases long. This is of particular importance because most of the transcriptional information used for annotation comes from ESTs that typically cover only a few hundred bases, or from incomplete cDNA sequences. Moreover, while coding sequences are spliced and exons junctions can be precisely delimited by short ESTs this cannot be done for long unspliced 3^′^UTRs. In addition, while coding sequences have a reading frame that provides useful information for a correct annotation, there is not such a thing for 3^′^UTRs. Until this moment, the position of the PAS was usually determined by clustering ESTs finishing at the same position and examining for the presence of a polyA tail, not encoded in the genome. In addition, the presence of the canonical HexM approximately twenty bases upstream of the PAS was usually considered a good indicator of a bona fide 3^′^end although we now know that less than 60% of the PASs are associated with a canonical HexM [9,10].

From an evolutionarily point of view, however, previous studies also showed that PASs can be conserved between mice and humans [29] and among different vertebrates [30]. In this study, we observed that the drop in genomic conservation after the PAS can be used to identify not annotated 3^′^ends. Interestingly, this change in conservation was not observed for TSSs, possibly due to the strong evolutionary constrains acting upon 3^′^UTRs [18]. In addition, different evolutionary forces shape the proximal regions of PASs and TSSs. For example, 5^′^UTRs are flanked by promoters that contain transcription factors binding sites which are necessary to recruit the transcriptional machinery to the TSS. On the contrary, apart from the DSE necessary for a correct cleavage reaction, there are no other well-described conserved elements crucial for the 3^′^end processing of the messenger. Consistent with this view, Xie and colleagues [31] showed that most conserved elements in 5^′^ regions of genes are associated with binding sites for transcription factors, which usually fall outside the mature transcript, while most conserved elements in the downstream region correspond to AU rich elements and binding sites for miRNAs, which fall inside the mature transcript.

In this study, we used ESTs to define not annotated PASs that in general did not overlap with nearby-annotated gene. Thus, they were not direct evidences of a physical link between the proximal annotated gene and the predicted extended 3^′^UTR. However, the CDI value of the PTEs depended on whether they were putative PASs or TSSs, as shown in Figures 1 and 2. In particular, the distribution of the putative PASs resembled the distribution of the annotated PASs. This observation strongly suggests that at least some of these sites corresponded to the 3^′^ end of annotated genes. In order to provide evidence for a physical link between the annotated genes and their putative extensions, we examined the existence of orthologous transcripts using the predicted distal PAS. We found that for higher CDIs there was an increased probability of finding a transcript from other species supporting the predicted PAS. This result indicates that using this conservation parameter can facilitate the identification of bona fide 3^′^UTR extensions.

To look for more evidence of a physical link between the predicted extensions and the annotated gene, we then coupled the transcriptional data from other species together with deep sequencing reads from total RNA of different human tissues. Given the elevated number of short tags generated with this technique, it is now possible in many cases to reconstruct almost the entire 3^′^UTR of highly expressed genes. The brain specific isoform of the *ADD2* gene, with a 3^′^UTR of more than 6 Kb, is an example. Although the read-coverage is generally not complete for genes expressed at lower levels these gaps could potentially be filled by increasing the sequencing depth. Importantly, the signal-to-noise ratio of this method allowed us to detect a clear transition in the RNA signal for both high and low expression transcripts.

Although overlapping deep sequencing reads can reconstruct a putative extended 3^′^UTR, the possibility in which the locus being considered encodes for different overlapping transcripts not physically linked still exist. Using Northern blot analysis, we showed that this was not the case for two PTEs. In both cases, we observed a high molecular weight band that corresponded to the predicted isoform with an extremely long 3^′^UTR. We previously provided evidence of the existence of high molecular weight molecules by Northern blot analysis for the not annotated *CPEB3* and *ADD2* isoforms with similar results [15,16]. Interestingly, we found other examples in the literature, such as the *TNR* or *RORB* genes, where very high molecular weight bands were detected by Northern blot for their rodent orthologs [32,33]. These isoforms are longer than the annotated isoforms but they are consistent with the usage of the predicted PTEs identified in this study. Moreover, the majority of the isoforms that we predicted were abundantly expressed and might correspond to the most representative transcripts, if not the only one, used by these genes in specific tissues. In recent updates of the human datasets we noticed the incorporation of new lincRNAs that partially overlap and share the same 3^′^end of our predicted 3^′^UTR extensions. We therefore speculate that some of these lincRNA may be part of the 3^′^UTR of the immediately upstream gene, as we have experimentally shown here for the KCNB1 gene (Figure 6). To evaluate this possibility, Northern blot analyses are required to evaluate each case.

Comparative analysis using genomic and transcriptomic information from different species revealed that gene structure can be highly conserved across vertebrates [20] and could be used to improve gene annotation [34]. This observation, coupled with the presence of evolutionary conserved genomic signatures and high-throughput transcriptomic data, facilitated the design of novel approaches that can be used to improve gene annotation of extensively curated databases. Indeed, in this work, we identified a few hundred conserved PASs not represented in the current Ensembl human predictions, possibly the most exhaustive annotation of the human genome. Given that the identification of the PASs is strongly based on conservation across species, we rationalized that the methodology could be applied to other vertebrates. Therefore, we extended our analysis to rats and dogs using total RNA-Seq data recently deposited on the public archives. The annotation of the rat genome is on an advanced state because the species has been extensively used as animal model. Still, we detected several hundred conserved 3^′^UTRs not present on the current databases. The annotation of the dog genome is instead at an early stage. Thus, we found thousands of genes with distal PASs not represented on the existent models. Since the approach could be applied to different mammals, we propose that the method can be incorporated to the annotation pipeline of any species in the phylum. Moreover, we believe that the validation of the predictions in different organisms using species-specific transcriptional data will increase the confidence of the orthologous models.

The RefSeq and Ensembl databases, among others, are intended to provide a common framework for genome wide analyses, facilitating the communication of different laboratories around the world. However, a recurrent result from high throughput sequencing studies is that a considerable amount of transcribed elements map to not annotated regions, including untranslated regions of genes encoding for proteins [4,5]. For example, this was observed in an early study that investigated the transcripts bound by the RNA binding protein Nova, previously known to participate in the regulation of alternative splicing [35]. Unexpectedly, the authors found that many binding sites fall within 3^′^UTRs or a few hundred bases downstream of the annotated PASs, presumably on not annotated 3^′^UTR extensions. Interestingly, NOVA2, one of the two members of the Nova family, appears to have a highly conserved not annotated 3^′^UTR itself (data not shown). Similar results were recently obtained by another group studying the RNA binding protein TDP-43 [36].

Different cis-acting elements present in 3^′^UTRs can be recognized by RNA-binding protein and/or small RNAs. The effect of some of these trans-acting factors has been demonstrated using high throughput techniques. In particular, it has been elegantly shown using genome wide microarrays that variations in the levels of a miRNA change the stability of the population of mRNAs containing the specific recognition motif for that particular small RNA [37,38]. Importantly, many of these regulatory factors have subtle effects upon their targets. Therefore, the best way (and possibly the only one) to study their function is by analyzing their overall impact on the transcriptome. A better annotation of the 3^′^UTRs will facilitate the identification of all the potential targets for a particular regulator. This, in turn, will lead to an increase in the signal-to-noise ratio of the effect of the factor at a genome wide level.

## Conclusions

The present manuscript examines the current state of gene annotation in reference databases and, in particular, aims to improve the annotation of conserved 3^′^UTRs. The methods presented here can be used to facilitate the identification of not-yet-annotated bona fide 3^′^UTR extensions in vertebrates. We provide evidence that many conserved 3^′^ends are not represented in the commonly used databases, and we also make available a list of a few hundreds manually curated 3^′^ends not annotated in the rather exhaustive Ensembl data set. We believe that the application of these methods will increment the confidence of the orthologous models.

We envision that a more complete annotation of the genome will facilitate the analysis of the data obtained from high throughput studies. The work will be of particular benefit to the growing number of investigators using total RNA sequencing and CLIP techniques.

## Methods

### Identification of putative PTEs

All databases used in this study were downloaded from the UCSC genome browser [21] or the Ensembl web site. The RefSeq and Ensembl databases were downloaded on March, 2012 or later. ESTs with an extreme at the same chromosomal position or at one nucleotide of distance from one another were clustered. The number of ESTs that gave origin to a cluster is referred as the cluster support. In order to account for the few nucleotides variability associated with PASs, when several clusters fell at a distance of less than 25 nucleotides, only the most distal cluster was considered. The ESTs orientations were not considered in the analysis. Only PTEs with a cluster support of 4 or more ESTs were considered. PTEs falling inside exons of the reference database (RefSeq or Ensembl) were not considered either. However, PTEs inside the reference introns were included in the analysis as they might correspond to the TSSs of alternative promoters or to PASs of alternative last exons. PTEs that might correspond to 3^′^ or 5^′^ splice sites (SS) of not annotated exons were also excluded from the analysis. To this end, PTEs falling 25 nucleotides apart from any SS of a spliced EST were filtered out.

### CDI calculation

PhyloP scores [19] were used to obtain the CDI of transcripts’ extremes. To calculate the conservation of the mature transcript region, an interval of 50 nucleotides starting 50 nucleotides inside the mature transcript and finishing at the 3^′^ (or 5^′^) end of the mature transcript was considered. The conservation of the mature transcript region was defined as the arithmetic media of the PhyloP score of each nucleotide of the interval. To obtain the conservation of the proximal region, the average conservation score for the 50-nucleotide interval starting 50 nucleotides outside the mature transcript region was calculated. The CDI was calculated subtracting the conservation of the proximal region to the conservation of the mature transcript region (see Figure 1 for a summary).

### Determination of PTEs putative identity

In order to classify the PTEs in putative PASs or TSSs, the orientation and distance of the closest reference gene was considered. If the closest reference gene was transcribed towards the PTE, the PTE was classified as a putative PAS. If the closest reference gene was transcribed in the opposite direction, the PTE was classified as a putative TSS. The PTE and the closest transcript had to be less than 1 Mb apart from each other. PTEs that fell in genomic regions with no proximal reference genes were not considered in the analysis. If a PTE fell inside the intron of a reference gene, it was classified according to the orientation of the transcripts in which it was included. In the case of overlapping genes with opposite directions the identity of the PTE was undetermined and it was not further considered. See Additional file 1: Figure S1 for a summary.

### FDR estimation

To determine whether a putative PAS obtained analyzing one database (“examined database”) was annotated as a PAS on other database (“validating database”), all transcripts in the validating database that had a PAS in the same position as the PTE were considered. Additionally, these transcripts had to share their last intron with any intron of any gene on the examined database. A putative PAS was considered to be supported by a validating database if any of its transcripts satisfied these conditions.

In order to estimate the FDR, we considered all putative PASs with a CDI or CD, depending of the method, higher than a given cutoff value. Next, we determined whether these putative PASs were supported or not by a validating database. The FDR was estimated as the fraction of not supported putative PASs.

### Determination of the usage of conserved PASs using deep sequencing data

In order to evaluate the functionality of putative PASs used in other species, TransMap transcripts [20] from different vertebrates were analyzed. A TransMap transcript and an Ensembl gene were considered to share a similar structure if they had the same last intron. A TransMap transcript was considered to have an extended last exon if it shared a similar structure with an Ensembl gene but had a more distal PAS. For each of the transcripts with an extended last exon, an upstream interval spanning the transcript extension and a downstream interval spanning a genomic interval of the same length downstream of the putative PAS were defined (see Figure 4A). The deep reads data generated by Wang and colleges [2] and mapped to the human genome by the group of Dr. Guigó were downloaded form the UCSC genome browser. The 8 tissues/organs considered were adipose, brain, breast, colon, heart, liver, lung and muscle. Each base in the interval was classified into two mutually exclusive categories: covered by a read or not covered. The orientation of the reads was not considered. All the covered bases in the intervals were counted and divided by the length of the intervals times a hundred to obtain the percentage covered of each of the intervals. The coverage difference was defined as the subtraction of the coverage percentage of the downstream interval to the coverage percentage of the interval upstream of the PAS.

The same method was used to identify conserved PASs in dogs and rats. The following deep sequencing runs were used: SRR388734, SRR388736, SRR388737, SRR388738, SRR388742, SRR388743, SRR388745, SRR388746, SRR388749 and SRR388754, and SRR042499 [39]. The reads were aligned to their respective genomes using Bowtie [40]. Reads mapping only once to the genome and with at most 2 mismatches were considered for subsequent analysis. The reads were visualized using IGV [41].

### Northern blot analysis

Northern blots analyses were performed using standard procedures. Briefly, for the *Kcnb1* and *Kcnq3* mRNA analysis, 5 μg and 25 μg respectively of mouse total RNA from brain, testes and heart were used. The samples were run in a 1.2% denaturating agarose gel at 70 V for 5 hours. The RNA was transferred to a nitrocellulose membrane by capillarity, UV cross-linked and pre-hybridized at 65°C for half an hour. The probes used were amplified from mouse genomic DNA with the following set of primers: 5^′^ CCAGTCTCAACCCATCCTCAA 3^′^ and 5^′^ GTCATCAGTGTCGGTGTCTA 3^′^ for the *Kcnb1* probe, and 5^′^ AACTGGACTTCCTCGTGGACA 3^′^ and 5^′^ CATGGAACCACTGGGTGTGAA 3^′^ for *Kcnq3* probe. For each probe, the unique amplified band was then purified from gel and radiolabeled using [α-^32^P]dCTP. The identity of the band was verified by sequencing. Hybridization was performed overnight at 65°C and the next day the membrane was washed twice with SSC 2X, SDS 0,1% and once with SSC 1X, SDS 0,1%. The membrane was then exposed ON to a pre-blanked Cyclone screen.

## Abbreviations

UTR: Untranslated region; mRNA: messenger RNA; PAS: Polyadenylation site; HexM: Hexanucleotide motif (polyA signal); DSE: Downstream sequence element; miRNA: micro RNA; EST: Expressed sequence tags; CDI: Conservation drop index; TSS: Transcription start site; PTE: Potential Transcript extremes; FDR: False discovery rate; CD: Coverage difference; ON: Overnight; EtBr: Ethidium bromide.

## Competing interests

The authors declare that they have no competing interests.

## Authors’ contributions

AFM and MM designed the study, interpreted the data and wrote the manuscript. MM performed most of the experiments. MM and AI performed the Northern blots. All authors read and approved the final manuscripts.

## Supplementary Material

Additional file 1: Figure S1 Describes the criteria used to classify PTEs in putative PASs or TSSs. Click here for file

Additional file 2: Table S1 Lists the putative PASs identified with high CDI. Click here for file

Additional file 3: Figure S2 Depicts two examples of PTEs with high CDI and no support from other databases. Click here for file

Additional file 4: Figure S3 Shows the correlation between CDI values calculated using the Mammalian and Vertebrates PhyloP scores. Click here for file

Additional file 5: Table S2 List the putative PASs identified with CD higher than 50% in humans. Click here for file

Additional file 6: Table S3 List the putative PASs identified with CD higher than 50% in rats. Click here for file

Additional file 7: Figure S4 Examples of PTEs identified in rats. Click here for file

Additional file 8: Table S4 List the putative PASs identified with CD higher than 50% in dogs. Click here for file

Additional file 9: Figure S5 Examples of PTEs identified in dogs. Click here for file

Additional file 10: Figure S6 Homolog PTEs of the Kcnb1 gene in rats. Click here for file

Additional file 11: Figure S7 Homolog PTEs of the Kcnq3 gene in dogs. Click here for file
