# Supplementary figures and images for: Identification of 3′ gene ends using transcriptional and genomic conservation across vertebrates
Source: BMC Genomics. 2012 Dec 18;13:708. doi: 10.1186/1471-2164-13-708 (PMC3564943; doi:10.1186/1471-2164-13-708)

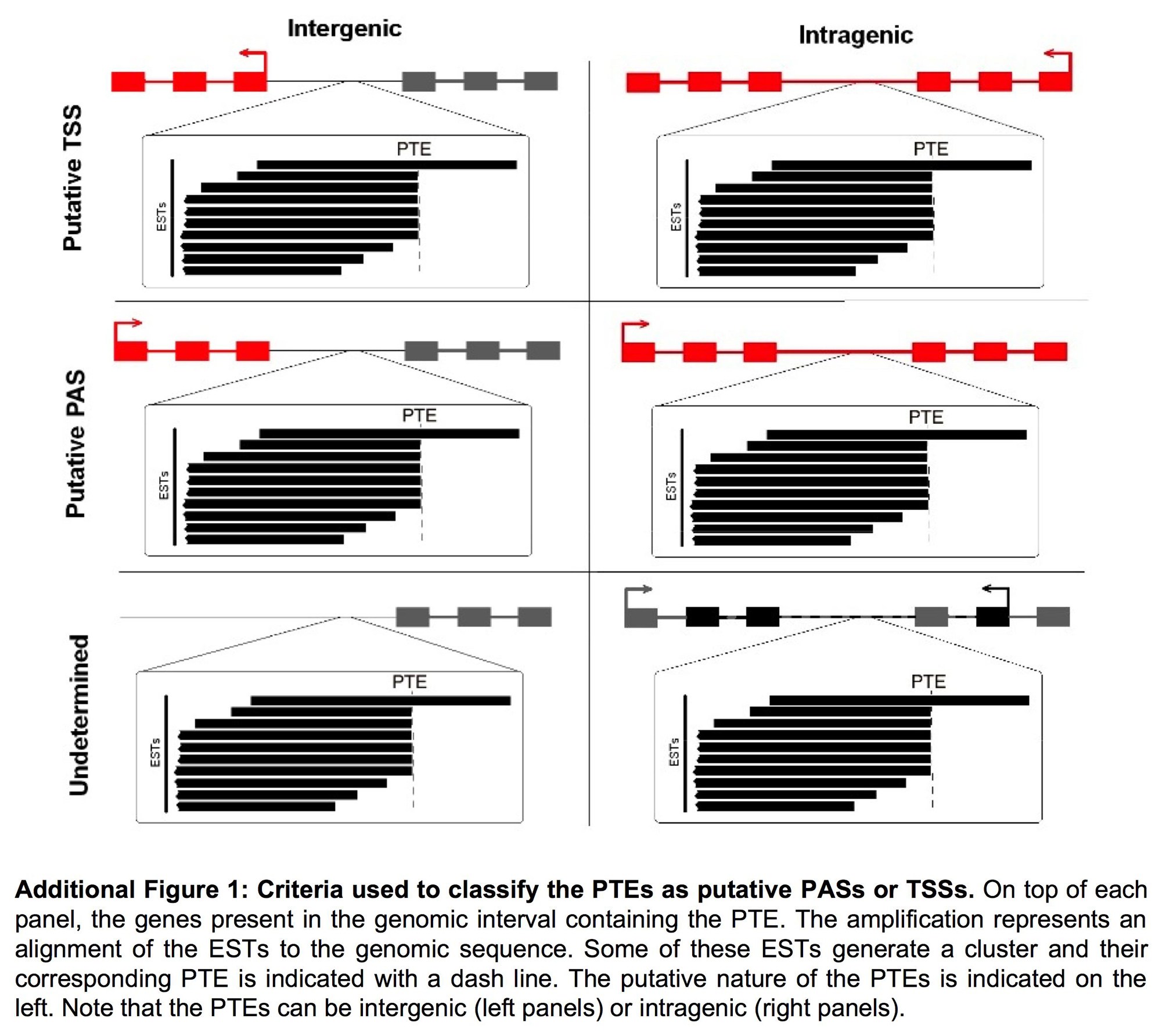

Supplement: Additional file 1: Figure S1 — Describes the criteria used to classify PTEs in putative PASs or TSSs. [file 1471-2164-13-708-S1.jpeg]

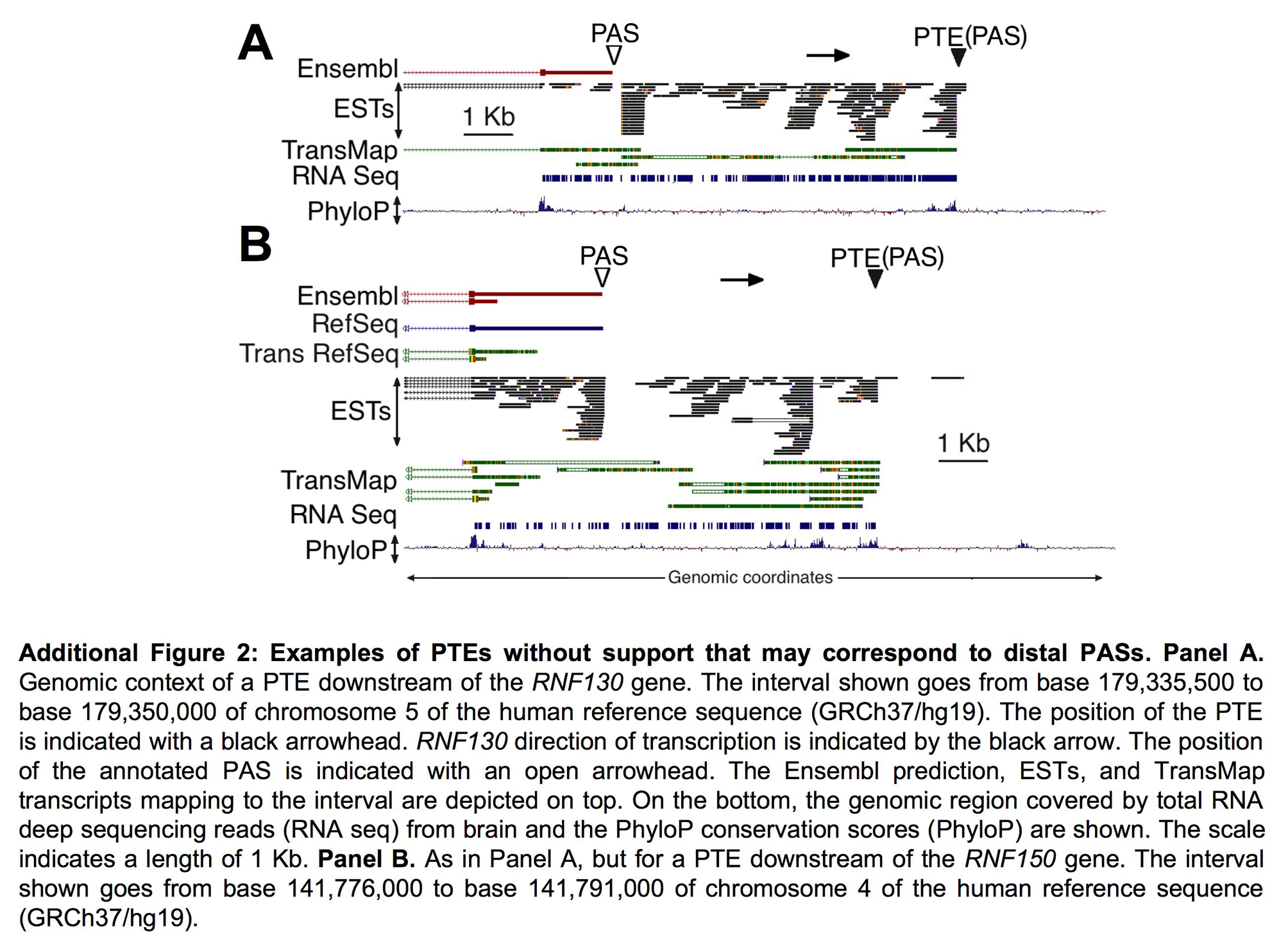

Supplement: Additional file 3: Figure S2 — Depicts two examples of PTEs with high CDI and no support from other databases. [file 1471-2164-13-708-S3.jpeg]

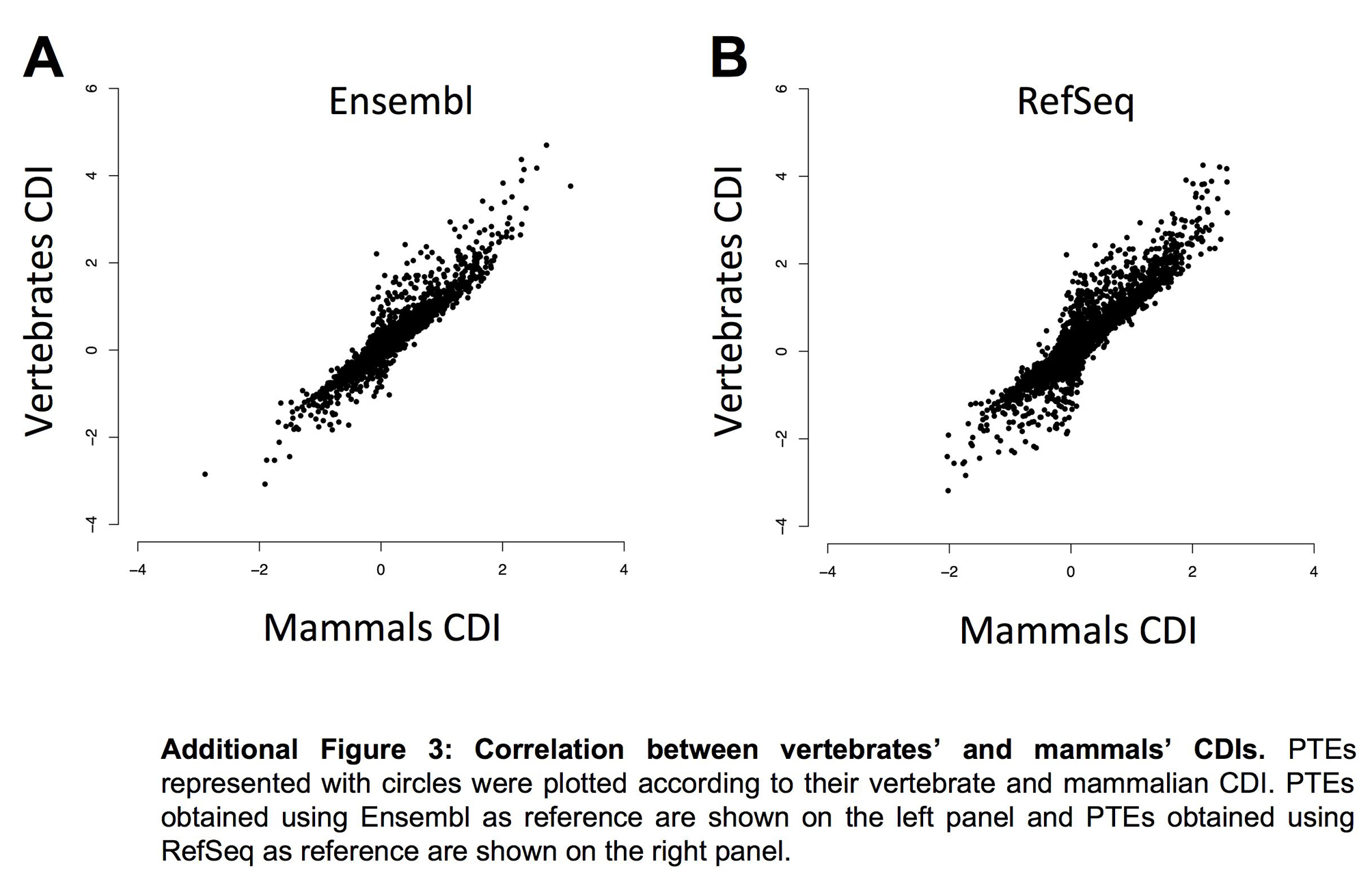

Supplement: Additional file 4: Figure S3 — Shows the correlation between CDI values calculated using the Mammalian and Vertebrates PhyloP scores. [file 1471-2164-13-708-S4.jpeg]

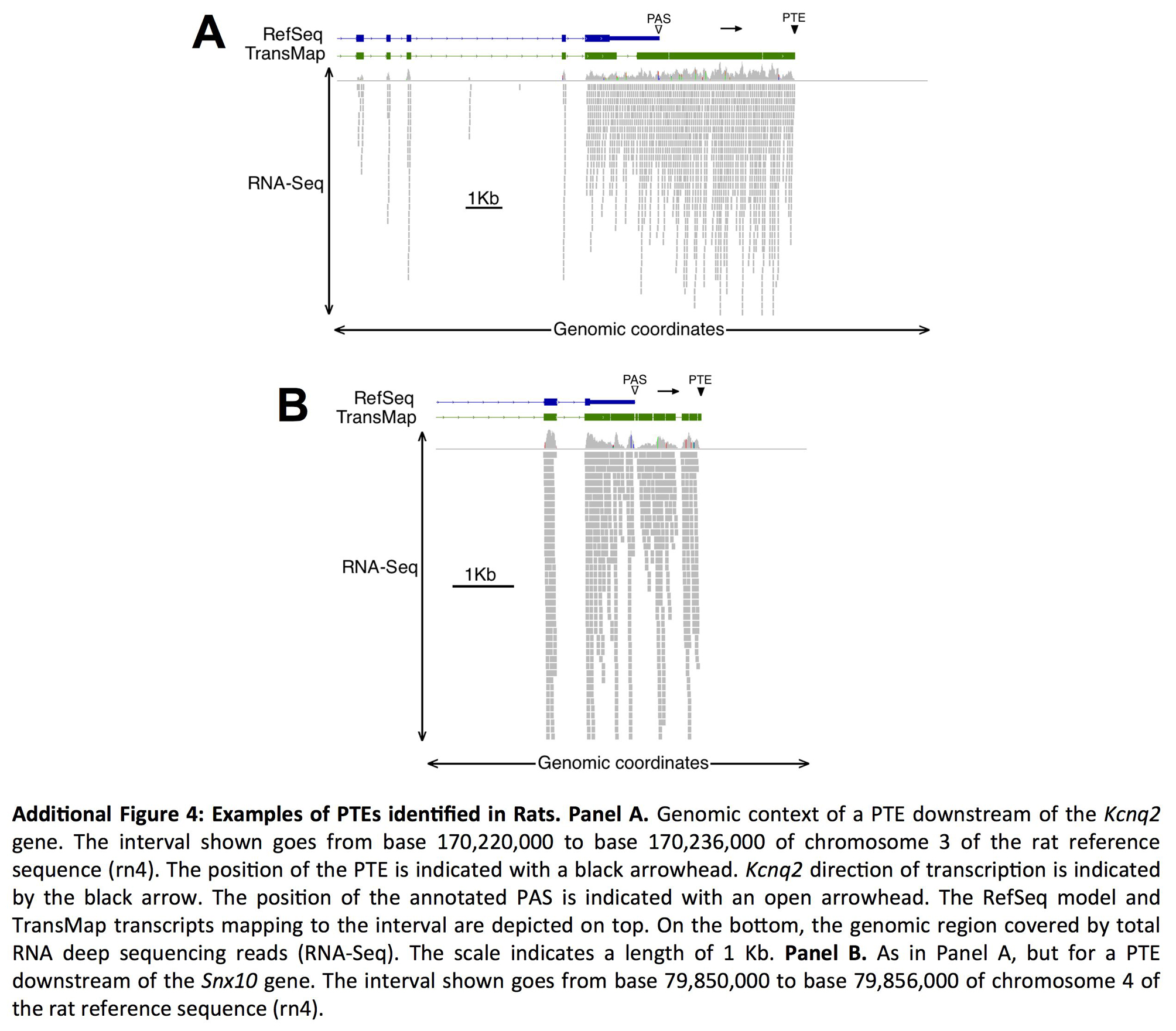

Supplement: Additional file 7: Figure S4 — Examples of PTEs identified in rats. [file 1471-2164-13-708-S7.jpeg]

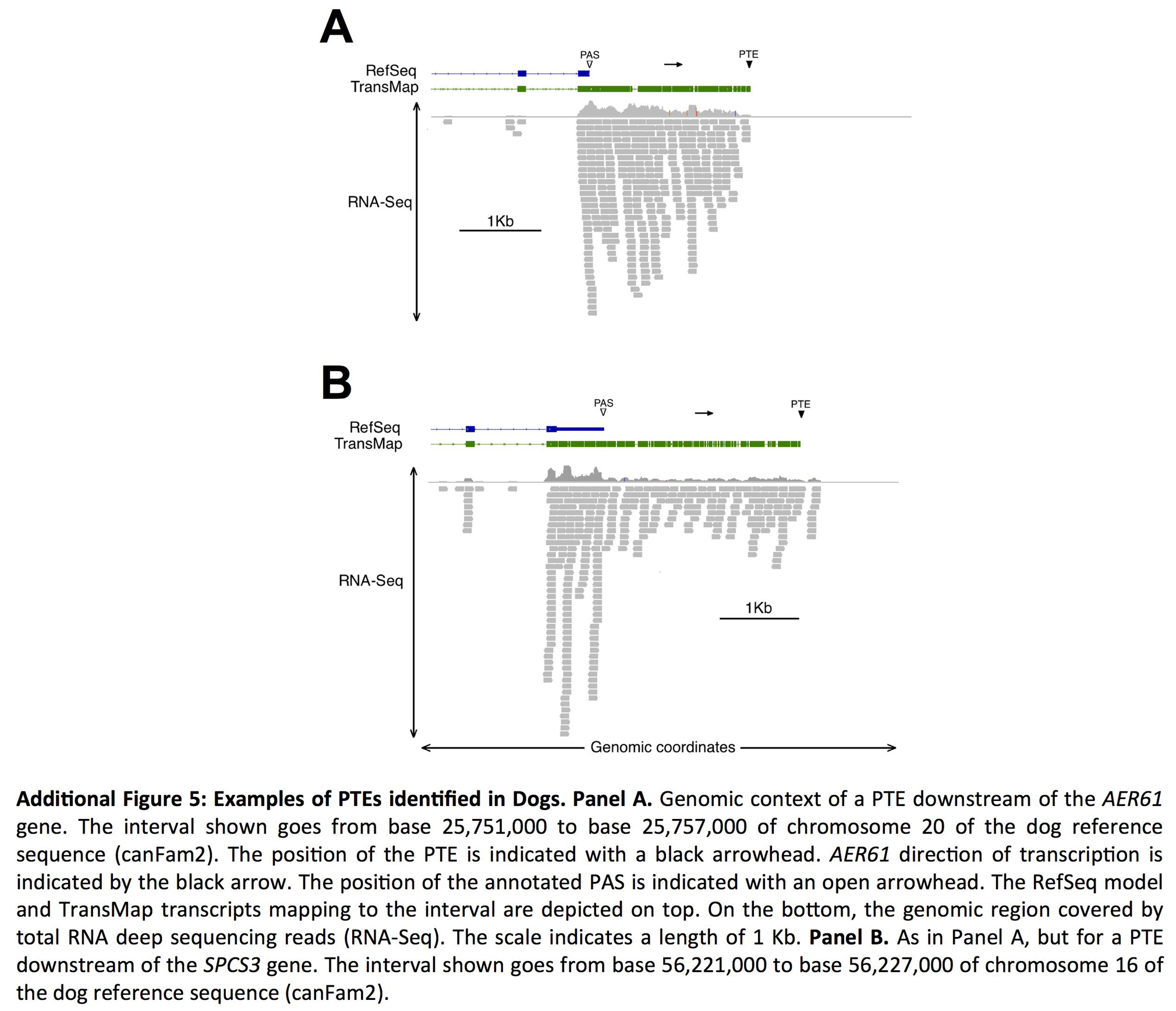

Supplement: Additional file 9: Figure S5 — Examples of PTEs identified in dogs. [file 1471-2164-13-708-S9.jpeg]

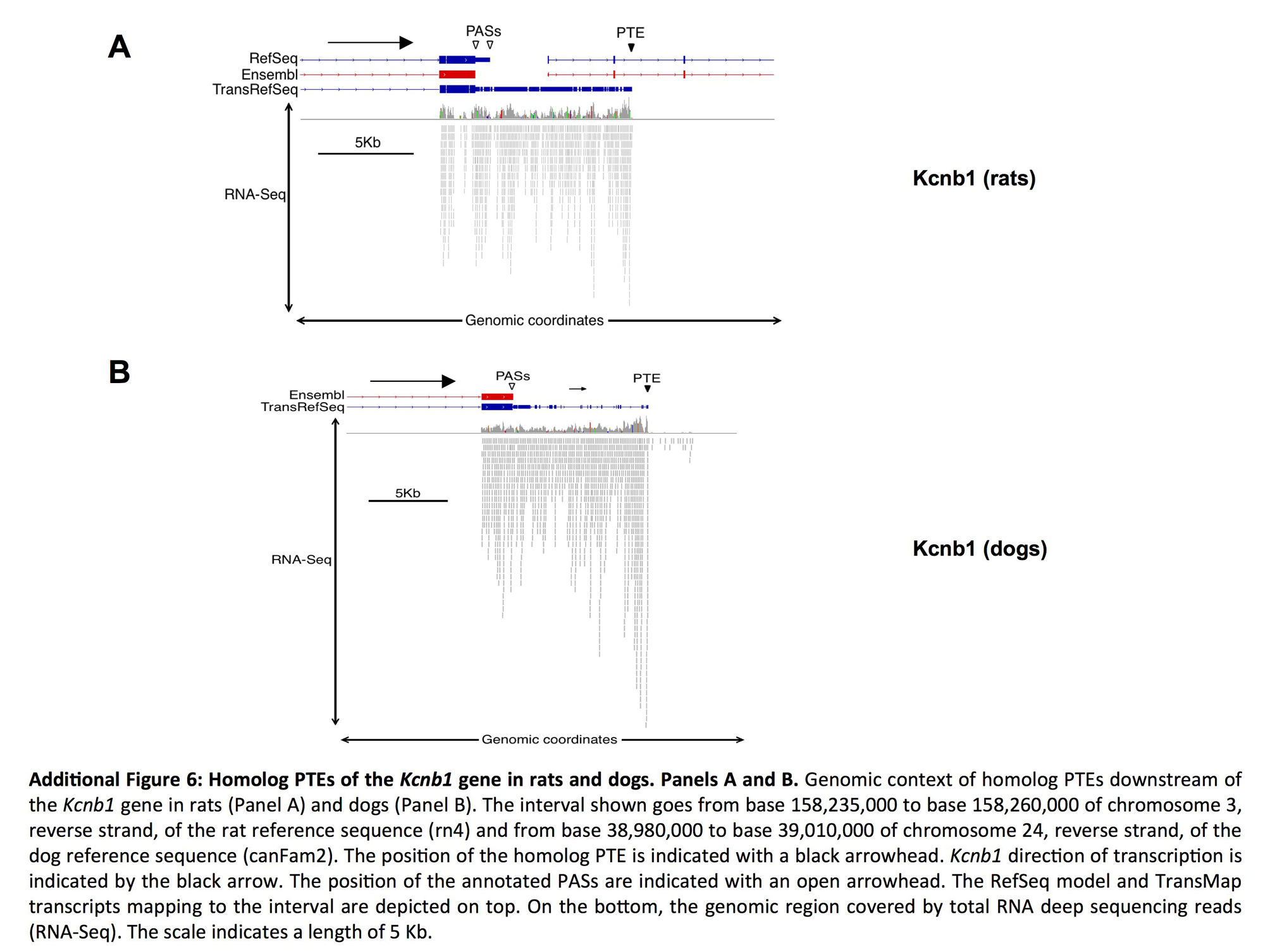

Supplement: Additional file 10: Figure S6 — Homolog PTEs of the Kcnb1 gene in rats. [file 1471-2164-13-708-S10.jpeg]

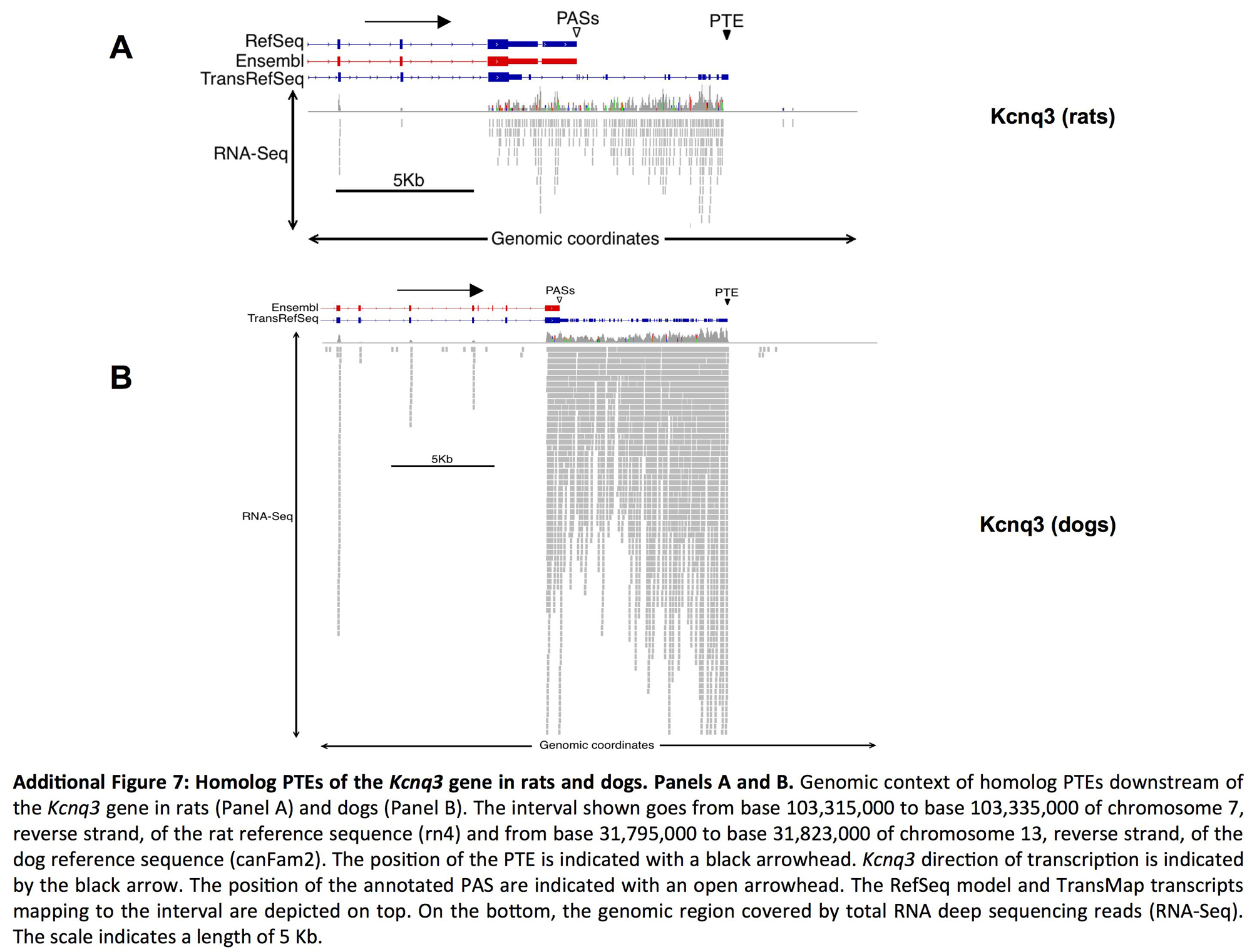

Supplement: Additional file 11: Figure S7 — Homolog PTEs of the Kcnq3 gene in dogs. [file 1471-2164-13-708-S11.jpeg]
